# Supplementary figures and images for: Superinfection exclusion creates spatially distinct influenza virus populations
Source: PLoS Biol. 2023 Feb 9;21(2):e3001941. doi: 10.1371/journal.pbio.3001941 (PMC9910727; doi:10.1371/journal.pbio.3001941)

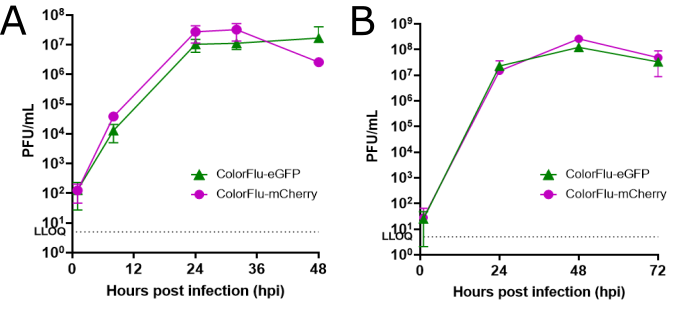

Supplement: S1 Fig — (A) Single cycle growth kinetics of ColorFlu viruses were assessed by infecting MDCK cell monolayers at an MOI of 2.5 PFU/cell and harvesting the supernatant at the time points indicated. Virus titre was assessed using plaque assay on MDCK cells. (B) Multi-cycle growth kinetics of ColorFlu viruses were assessed by infecting MDCK cell monolayers at an MOI of 0.001 PFU/cell and harvesting the supernatant at the time points indicated. The mean and SD are shown (n = 3). For all time points in A and B, the titres of ColorFlu-mCherry and ColorFlu-eGFP were not significantly different (Mann–Whitney U test, p > 0.05). LLOQ = Lower limit of quantification. Underlying data can be accessed at the following address: http://dx.doi.org/10.5525/gla.researchdata.1370. (TIF) [file pbio.3001941.s001.tif]

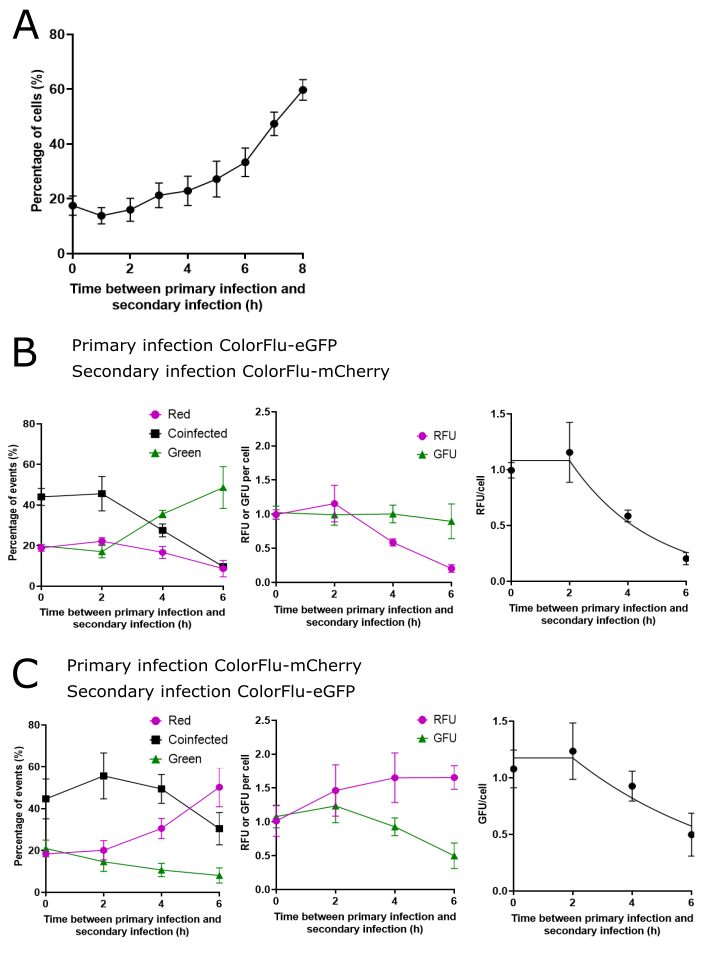

Supplement: S2 Fig — (A) Percentage of cells that were negative for both mCherry and eGFP expression, determined by flow cytometry. MDCK cells were infected with Colorflu-eGFP before secondary infection at the time points indicated with ColorFlu-mCherry, with both viruses at MOI 1 FFU/cell. Data presented as mean and SD (n = 6). MDCK cells were infected with either (B) Colorflu-eGFP or (C) ColorFlu-mCherry before secondary infection at the time points indicated with the other virus, with both viruses at MOI 1 FFU/cell. The percentage of fluorescent cells was then assessed using flow cytometry. The number of red and green forming units per cell (RFU, GFU) was calculated from the percentage of red, green, and coinfected cells under the assumption that infection follows a Poisson distribution. The number of secondary viruses detected per cell were used to fit a model in which the number of secondary viruses per cell that could be detected was constant for 2 h and then decayed exponentially to zero with increasing intervals between infections. The SST for the models in (B) and (C) are 0.22 and 0.24, respectively. Data are presented as mean and SD (n = 3). Underlying data can be accessed at the following address: http://dx.doi.org/10.5525/gla.researchdata.1370. (TIF) [file pbio.3001941.s002.tif]

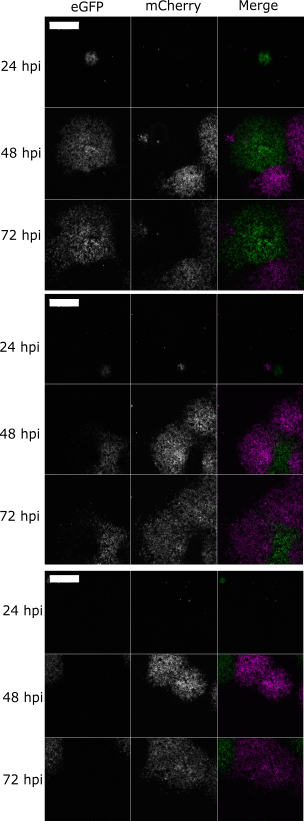

Supplement: S3 Fig — Viruses were seeded onto monolayers of MDCK cells, overlayed with agarose, and imaged every 24 h. Images taken on Celigo fluorescent microscope. Scale bar = 2 mm. Underlying data can be accessed at the following address: http://dx.doi.org/10.5525/gla.researchdata.1370. (TIF) [file pbio.3001941.s003.tif]

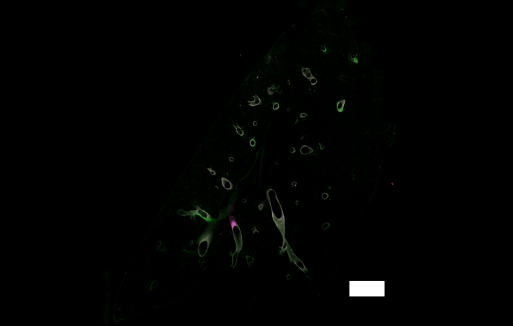

Supplement: S4 Fig — C57BL/6 mice were intranasally inoculated with mixtures of mCherry and eGFP expressing ColorFlu viruses (500 PFU of each virus). Lung sections, taken at 3 dpi, were imaged using a Zeiss LSM 800 with a 20× objective lens. Scale bar = 1,500 μm. Underlying data can be accessed at the following address: http://dx.doi.org/10.5525/gla.researchdata.1370. (TIF) [file pbio.3001941.s004.tif]

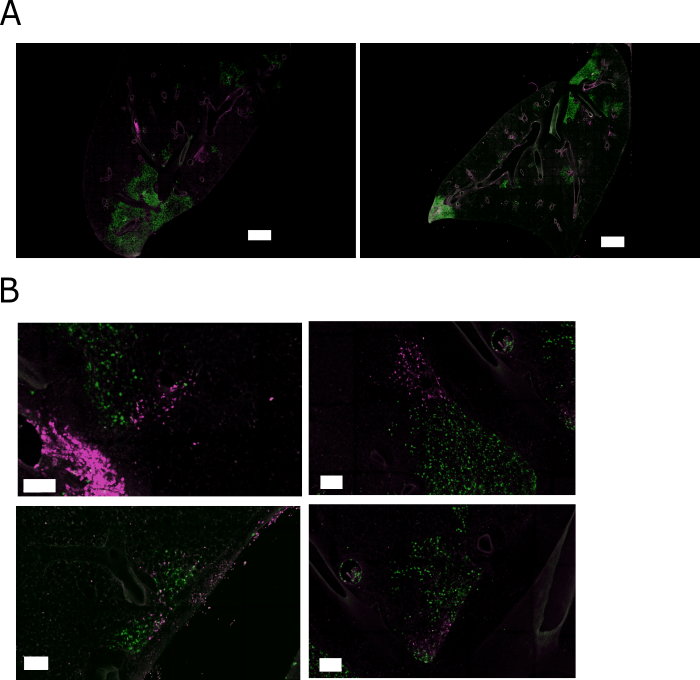

Supplement: S5 Fig — C57BL/6 mice were intranasally inoculated with mixtures of mCherry and eGFP expressing viruses (500 PFU of each virus). Lung sections, taken at 6 dpi, were imaged with a Zeiss LSM 800 using a 20× objective lens. (A) Confocal micrographs of whole lung slices from infected mice 6 dpi. (A) Whole lung images. Scale bar = 1,500 μm. (B) Enlarged images of infected lesions. Scale bar = 100 μm. Underlying data can be accessed at the following address: http://dx.doi.org/10.5525/gla.researchdata.1370. (TIF) [file pbio.3001941.s005.tif]
