# Supplementary material for: Superinfection exclusion creates spatially distinct influenza virus populations
Source: PLoS Biol. 2023 Feb 9;21(2):e3001941. doi: 10.1371/journal.pbio.3001941 (PMC9910727; doi:10.1371/journal.pbio.3001941)
Supplement: Attachment — Submitted filename: Sims et al Response to Reviewers.docx [file pbio.3001941.s007.docx]

**Response to reviewers**

We would like to thank the reviewers for their comments on our manuscript. We were extremely pleased that the reviewers recognised the importance of the study and that they considered the work overall to be ‘elegant,’ ‘rigorously performed’ and of ‘broad interest’ to ‘the influenza community, and virologists in general.’ We also welcome the opportunities to improve the manuscript that this review process has allowed us, in particular by clarifying its focus and improving the detail in our model of SIE kinetics. We appreciate the very clear editorial statement that accompanied the reviews, which has helped to direct our response.

In response to the reviews we have extensively revised our manuscript, condensing and re-structuring the text to strengthen the framing and focus the writing in a way that we hope will make the novelty and broad applicability to other viruses clearer to non-influenza readers.

In response to points raised by reviewers #1 and #2, we have repeated our flow cytometry experiments with additional time points. This led us to refine our model to more accurately reflect the kinetics of SIE. In our opinion this experiment has substantially strengthened the paper and we thank the reviewers for this valuable suggestion. We have also clarified how we performed our calculations and carried out additional control experiments as requested, including reversing the order of addition of our viruses. All of these changes are described in a point by point response the reviewers, which follows below.

We are pleased to be able to submit a revised manuscript that in our opinion has been significantly improved by the review process, and we hope it will be considered suitable for publication in *PLOS Biology*.

Responses to Reviewer 1

1. *It isn't clear from the data presented that the eGFP and mCherry viruses have similar fitness. One step growth curves are not particularly sensitive (Supp Data 1) and it appears that they do have differences in single cycle. Suggest either a competition assay or controlling for potential issues by reversing the order of viruses in key experiments (e.g. mCherry then eGFP or eGFP then mCherry) to make sure these apparent growth differences don't affect the results and interpretation. For example, in Figure 1B, mCherry single infected cells seem to be much greater than eGFP single infected cells when introduced together. Flipping the order in this experiment would be helpful.*

Following the reviewer’s suggestion, we performed the experiment of flipping the order of the viruses, carrying out infections under the same conditions but with the order of addition of the viruses changed (Supplementary Figs 2B, C). The kinetics of SIE for both orders were a good fit our model, with small variations in the fitting parameters. However, the reviewer was correct that the results were not identical – this can be most clearly seen by comparing the degree of SIE at 6 h post primary infection. This is interesting, given that the only difference between the viruses is the fluorophore they encode, and that the fitness of the viruses, as assessed by multicycle replication kinetics, are extremely similar (Supplementary Figure 1B). Why the parameters of SIE are slightly different may be relevant to future studies that aim to uncover the mechanism of SIE in influenza viruses. However, it does not change the overall argument of the present paper, and we are glad to be able to include experimental data that makes this point (discussed in lines 178 – 182).

2. *Although I am inclined to agree that viral replication underlies the mechanism of SIE, wouldn't there be other mechanisms that could lead to exponential changes other than viral replication itself? (For example cell intrinsic immune cascades? I do realize that Sun and Brooke found it appeared to be interferon independent)*

The main argument of this paper considers the effects of a delayed onset of SIE and is agnostic about its mechanism. However, the reviewer’s comment here, and others, showed us that the focus of the paper should have been more clearly expressed. We have extensively reworded the paper to clarify this. Having said this, where we do discuss possible mechanisms we very much agree with the reviewer. We cite the work of Sun and Brooke (ref 42) which as they note suggests that SIE does not depend on innate immune signalling, and our own unpublished work is also consistent with this. However in the current study, although we show that SIE correlates with the timeframe of viral gene expression, we have not sought to establish if this is a direct effect or an indirect effect mediated by cellular factors. We have added discussion to clarify the scope of our current findings (lines 166-170 and 374-391).

3. *Supp Data 1 - In single cycle growth curve, which is arguably more important here given time frames employed in experiments, the mCherry virus does appear to have an advantage over the eGFP one up to 30 hours. It does not appear that statistics were done on panel A, only B? Also, number of replicates should be specified (e.g. points per time point). Unclear why MWU test used rather than t-test (are they non-normally distributed)? This has relevance to assessment of relative fitness of the viruses.*

Although it did appear from the data that mCherry might have a growth advantage over a single round of infection up to 30 hpi, the difference is not statistically significant. The reviewer’s earlier suggestion, of flipping the order of infection to assess whether any differences between the viruses were relevant to our model, was helpful in showing that this was not a concern for our argument (Supplementary Figs 2B, C; see above). Statistics were performed on both panels but this was not sufficiently clear in the first submission; we have reworded the figure legend to make this clearer and to clarify the number of repeats. We chose the MWU test as a conservative choice: with N = 3 we could not convincingly assess normality, so we chose a non-parametric test.

4. *Line 63 - Suggest removing "therefore" from this sentence (used to two successive sentences)*

This line has been removed from the manuscript .

5. *Line 199 - should read Figure 2BC?*

This has been corrected.

6. *Line 203 - should read Figure 2B?*

This has been corrected.

7. Line 350 - should read "could be" instead of "could are" or simply "are"

This has been corrected.

Responses to Reviewer 2

1. *The authors comment on genome interactions throughout the manuscript, but do not show any evidence of genomic interaction in any of their experiments. The use of isogenic virus maintains replication rates of the 2 viruses but prevents the study of reassortment and rearrangement of viral genomes. To enhance this paper, the authors should generate viruses with trackable mutations that do not alter replication rates or capabilities but would allow for detection of genomic interactions and reassortments.*

On reading the reviewers’ comment we recognise that we may not have been sufficiently clear in outlining the scope of this study. Reassortment between strains is a particularly high-consequence result of coinfection, particularly from the perspective of pandemic emergence, but it is not the only genome interaction influenza viruses can undergo – other examples include the reassembly of error-free genomes through multiplicity reactivation, the modulation of viral gene expression and competition from defective interfering RNAs. We agree that having such a large portion of the introduction dedicated to reassortment had the potential to obscure the paper’s intended focus, which was to explain how SIE defines the regions in which coinfections (and hence genome interactions of any sort) could occur. We are pleased to have the opportunity to address this.

In the revised manuscript we have replaced the lengthy discussion of reassortment with a more concise and balanced discussion of different types of genome interaction (lines 33-40 and 323-334), and have added text to explain more clearly why an experimental design that looked specifically at reassortment between divergent strains would (as the reviewer notes) need to consider many aspects of functional compatibility between viral proteins, in addition to addressing SIE (lines 364-372). Finally, in response to this and other comments, we have rewritten the introduction and discussion extensively to focus the paper on what is interesting and important about the current set of results – namely that SIE controls the regions within which coinfection, and therefore genome interactions of any sort, can occur.

2. *In Figure 1, the authors state that SIE begins between 2 and 4 hrs post initial infection, but the evidence for this need to be expanded. Currently, the SIE at 4 hrs does not differ from 0 hrs between infections, so why do the authors assume SIE begins before then? The mathematical curve does show a beginning of a dip for RFU cells, but more timepoints particular between 2 and 6 hrs are needed to ensure the curve truly matches. Additionally, how the RFU and GFU are calculated needs to be further explained. Is this supposed to be red fluorescent units and green fluorescent units and measuring the intensity of fluorescence in each cell?*

This was an extremely helpful comment! In response to it we repeated our investigation of the timeframe of SIE with timepoints spaced at closer intervals (Figure 1). Doing so made it clearer that our original model – though correct in suggesting that SIE was initially minimal but then increased exponentially – did not fully describe the data. With closer time-sampling we were able to revisit the data and fit a model informed by our understanding of influenza virus biology, namely that a key transition in the viral replication cycle is the initiation of viral transcription after nuclear entry, and that this begins approximately 2 h post infection (see Dou *et al.* (2017) *Cell Rep*). We feel that doing so has significantly improved the paper and we greatly appreciate the reviewer’s suggestion that we do this.

We have also added an extended explanation of the calculation of RFU and GFU (lines 475 – 498).

3. *Figures 1 and 2 appear to confirm previous reports that 6 hrs between infections is the first timepoint SIE is observed without contributing new knowledge. Additionally, having more cells infected with the primary virus prevents the secondary virus about 30 min faster is what appears to be the only novel data here. Also, in the figure legend, it is stated that these experiments are done at an MOI of 0.5. From the images, it seems that more than 75% of the cells are infected suggesting a higher MOI*

While we do think that the modelling of coinfection kinetics in figures 1 and 2 adds something to our understanding of the topic (and have reworded our conclusions and discussion to emphasise this, lines 181-183; 200-203, 373-390) we agree with the reviewer that this part of the paper was a development of a topic that had already been studied. The reason we did this was to set up what is in our opinion the main novelty and interest of this study: to enable us to understand how SIE patterns interactions in spreading infections. This is the focus of the second half of the paper (Figures 3 and 4).

Regarding the MOIs, in the original submission these experiments were carried out using an MOI of 0.5 of each virus. This gave a total MOI of 1, which is why around 75% of the cells being infected. We have revised our phrasing to make this clearer. In addition, on repeating the experiment in Figure 1, we did use an MOI of 1 for each virus – this gave a total MOI of 2, resulting in around 85% of the cells being infected with one or both viruses.

4. *Line 348, How does the capability for reassortment contrast with your data? Data show that coinfection can occur during SIE conditions in cell culture and mice albeit at low percentages of cells. How many cells do you expect you would need for reassortment to occur? I would expect a few coinfected cells would be sufficient. Since isogenic viruses are used in this manuscript, there is no data on reassortment, and the discussion should acknowledge this fact.*

We agree with the reviewers’ points here, and realise that we may not have emphasised this clearly enough in our original submission. We have extended and rewritten our discussion of why reassortment would be observed in other studies despite the effects of SIE (lines 51 – 54 and 341 – 363) and, as noted above, we have clarified that we intentionally used an experimental design that did not directly address reassortment (lines 364-372).

5. *Line 364-367, there are no data in this manuscript regarding coinfection using different strains.*

This is correct and as noted above we are grateful for the opportunity to clarify the manuscript’s focus so that it is clearer to the reader that we are seeking to define the way in which SIE constrains areas where coinfections can occur, rather than specifically examining reassortment. As well as removing extraneous text that may have given a misleading focus, we have inserted a paragraph specifically acknowledging this point (lines 364 – 372).

6. *I am confused why DI-RNAs are brought into the discussion. If a DI-RNA is present in a lesion, wouldn't that inhibit the viral replication in that lesion and prevent spread of that virus to another lesion? Data and experiments shown do not address DI-RNAs, so why are these in the introduction and discussion?*

We would make a different prediction from the reviewer in this case – DI-RNAs are known to gradually take over replicating viral populations (for example in serial egg passage) and based on our data we would predict that if a DI-RNA ‘contaminated’ a lesion it would be able to propagate within that lesion due to trans-complementation with intact genomes at the lesion’s edge. However, this is a hypothesis that is yet to be tested, and lies beyond the scope of what we are trying to say in the current study. We agree with the reviewer that the extent of discussion about DI-RNAs was distracting and we have therefore reduced it significantly (lines 39 and 334).

7. *Line 52, DI-RNAs can be more than large internal deletions. Insertions, copy-backs, snapbacks, etc. can also produce DI RNAs.  Even a 1 nt deletion can produce a frameshift that knocks out an essential protein and produces a DI RNA.*

We agree with the reviewer that all of these genome types are defective in at least some viruses, but in light of their previous comment this section has been removed.

8. *Is supplementary figure 1 A+B truly single cycle vs multi cycle? Can the authors comment what's the rationale of these experiments if the goal is to compare growth kinetics.*

To ensure single cycle infection cells were infected at MOI 2.5 to ensure every cell was infected, acid washed to remove any virus that did not enter immediately and grown in the absence of trypsin to ensure that any nascent virus could not infect cells. (We also would predict that at this MOI single cycle kinetics would be ensured by SIE.) To allow multi-cycle infection cells were infected at low MOI (0.001) and allowed to propagate through cells in the presence of trypsin. These details are described in lines 449 – 457. The aim of these experiments was to test whether there were any significant differences in growth kinetics between viruses that would complicate the interpretation of Figures 1 and 2 – for further discussion of this point please see the response to reviewer 1, point 1 (above).

9. *Figure legend for 1A, better to state size of bar in pictures (1 uM?) and not the 64x objective.*

This has been added to the legend of Figure 1A.

10. *Fig. 2A For ease of reading, please write the ratios of Green to Read virus instead of*

The ratios have been added to the Figure 2A.

Responses to Reviewer 3

1. *The data don't appear to support the conclusion that there is "a progressive shift from a permissive to exclusionary state beginning around 2h post-primary infection" (Lines 168 and 381). Rather, the first statistically significant evidence of exclusion is seen at 6h and no exclusion is apparent at 2h.*

We agree that this could have been more clearly expressed. As well as rephrasing this, in response to this and other comments we also completely repeated the experiment to provide better time resolution (Figure 1). We can now show that the SIE in ineffective for intervals between infection of up to 2h, after which it increases exponentially (Fig 1D). This means that the proportion of coinfected cells decreases significantly for intervals between infection of 3h or greater (Fig 1D, lines 127-8, 144 – 146).

2. *Fig. 2: The measured RFU/cell declines with increasing dose of primary virus. This effect is seen consistently across replicates and time points but is not discussed. At the high MOIs used, limitations on cellular resources may result in interference within coinfected cells.*

This is an interesting observation. We agree with the reviewer that the mean RFU/cell values in Fig 2B prior to the onset of SIE (at intervals of 0 h and 2 h) consistently decrease with increasing doses of the primary (green) virus. However, these differences in the mean values are not statistically significant (p > 0.05 by Kruskal Wallis test), and their magnitude is very small compared to the much larger changes seen when the amount of secondary (red) virus is varied. We have replotted these data here to illustrate this point:

This is not to rule out that there could be differences in gene expression caused by the MOI of viruses at primary addition, but they are too small to confidently detect with the data we have, would be hard to interpret using this system (given that in such cases viruses of the same colour would also suppress each other), and if real would relate to interference with simultaneous infection rather than with superinfection, which was our focus in this case. We think that the reviewer’s hypothesis about limitations on cellular resources interfering with simultaneous coinfection is an interesting one, and it is something that we would be interested to test ourselves in the future. However, we do not feel that we have enough data to discuss it in our current study.

3. *Line 125: The description of SIE as rapid is subjective. Four hours is a lot of time in the life of an influenza virus.*

We agree that this is unclear and have removed this word ‘rapid.’

4. *Figure 2C: About 50% of cells are positive for green, but red is excluded from about 90% of cells. What might explain this?*

We assume that this may be due to a combination of effects, including the preferential loss of infected cells rather than uninfected cells from the analysis (now discussed in lines 159 – 162 and Supplementary Fig 2A), the presence of cells that are non-permissive to any infection, and the presence of SIPs or other viruses that are unable to express the encoded fluorophore. We note that the last point in particular may relate to the slight variations between ColorFlu strains that were described in the revised submission in response to reviewer 1 comment 1, above (Supplementary Figures 2B,C). However, although it is important for us to be mindful of the non-fluorescent cells so that we do not over-interpret our data, they should not change the parameters which describe the kinetics of SIE, and so should not alter the argument of the paper.

*5.            Discussion of how to reconcile SIE with data on reassortment in experimental systems overlooks a number of relevant published observations:*

*- reassortment is common even when coinfecting viruses are delivered through two independent transmission events (Tao et al. JVI 2015)*

*- reassortment is common and increases over time even when relatively low inoculation doses are used (e.g. 1000 PFU; Tao et al. JVI 2014)*

*- reassortment is common when co-infecting viruses are delivered to guinea pigs 12 h apart (Marshall et al. PPath 2013) Better explanations for abundant reassortment in the context of SIE include:*

**

*- the difference in tissue sites examined (nasal for reassortment; lung in the present work)*

*- The difference in virus strains used. PR8 replicates more quickly than most IAVs and is likely to induce SIE more rapidly. Multiple have been tested for reassortment and reassortment frequencies vary (Phipps et al. NatMicro 2020).*

*- differences in host species may affect kinetics of innate responses and spatial dynamics of viral dispersal*

We thank the reviewer for highlighting these papers and agree that they are relevant. We have discussed the papers noted (the references cited are 10, 11, 8, 19, respectively) as well as citing additional papers in the heavily revised and condensed introduction and discussion, notably in lines 41 -54 and 349-363. We do not consider our results at all incompatible with the highlighted publications, because our results imply that coinfection is more restricted than previously thought but not impossible. We are glad to have been able to clarify this point.

Minor comments:

1. *Line 2: check short title - perhaps a word is missing.*

The short title has been reworded.

2. *Line 66-67: Rephrase for clarity. 'Viral genomics' is vague and 'imply' does not convey well the absolute requirement of coinfection for reassortment. Perhaps "Detection of reassortant viruses in circulation indicates that cellular coinfection must occur with some frequency."*

We realised the point we were trying to convey with this line was repeated further down the paragraph and so we deleted it entirely.

3. *Line 72: Note that the frequency of coinfection would not by modulated by selection. This experiment shows that the frequency of cellular coinfection in this model host is sufficiently high to sustain viral replication over several days.*

Our original phrasing was misleading and we have corrected this (line 52-56).

4. *Fig 1A: please define the scale bar and add units to the MOI given in the figure legend.*

This has been corrected.

5. *Line 190: I suggest rephrasing this subtitle in light of the fact that only two parameters were tested - so a claim that the main determinant was identified seems to overstate things.*

We and have corrected this to read “The kinetics of superinfection exclusion are impacted by the dose of the primary infecting virus, but not the secondary infecting virus” (line 184-5)

6. *Line 195 and throughout: the effort to handle the data quantitatively is commendable, but IC50 doesn't seem to be the right term here in that the time interval leading to 50% inhibition, rather than a concentration, is estimated.*

We agree with the reviewer that using IC50 as an analogy was unhelpful. In response to other comments (above) we have substituted a different model, and doing so removed this terminology (lines 172 – 176).

7. Line 199: Figure 3 is referred to erroneously.]

This has been corrected.

8. Line 233: The reference to a 'switch' here suggests a binary state and may want to be reconsidered. The inability to overcome SIE with a 5-fold increase in the dose of the second virus makes sense if SIE is driven by a factor that accumulates exponentially (as suggested by the authors).

We agree and have corrected it to read “once established, the exclusionary state in the cells established by SIE would be hard to overcome.” (lines 202-3)

9. Line 285: "In a natural infection we assume that it is unlikely that multiple 'incoming' viruses would reach the same cell within a short space of time." This statement should be revised in light of the fact that essentially simultaneous co-infection would occur commonly as viruses budding out of one cell infect a neighboring cell.

We agree this statement was confusing – we were referring specifically to the point when viruses are establishing infection in a new host, which may not have been clear in the original wording. We have revised the manuscript to read “Outside of an experimental setting it is unlikely that two unrelated virus particles would reach the same cell at exactly the same moment. Instead, one would expect unrelated viruses to replicate locally within a host organism before eventually encountering the same cell.” (lines 55-7)
